# TLR signaling pathway and the effects of main immune cells and epigenetics factors on the diagnosis and treatment of infertility and sterility

**DOI:** 10.1016/j.heliyon.2024.e35345

**Published:** 2024-07-26

**Authors:** Kosar Babaei, Mohsen Azimi Nezhad, Seyedeh Nafise Sedigh Ziabari, Ebrahim Mirzajani, Hossein Mozdarani, Seyedeh Hajar Sharami, Sara Farzadi, Seyed Reza Mirhafez, Misa Naghdipour Mirsadeghi, Seyedeh Elham Norollahi, Zahra Saadatian, Ali Akbar Samadani

**Affiliations:** a Noncommunicable Diseases Research Center, Neyshabur University of Medical Sciences, Neyshabur, Iran; b UMR INSERM U 1122, IGE-PCV, Interactions Gène-Environment En Physiopathologie Cardiovascular Université De Lorraine, Nancy, France; c BSC of Midwifery, Reproductive Health Research Center, Al-Zahra Hospital, Guilan University of Medical Sciences, Rasht, Iran; d Department of Biochemistry and Biophysics, School of Medicine, Guilan University of Medical Sciences, Rasht, Iran; e Department of Medical Genetics, Faculty of Medical Sciences, Tarbiat Modares University, Tehran, Iran; f Reproductive Health Research Center, Department of Obstetrics and Gynecology, School of Medicine, Al-Zahra Hospital, Guilan University of Medical Sciences, Rasht, Iran; g Department of Gynecology, School of Medicine, Alzahra Hospital, Guilan University of Medical Sciences, Rasht, Iran; h Department of Gynecology, School of Medicine, Reproductive Health Research Center, Alzahra Hospital, Guilan University of Medical Sciences, Rasht, Iran; i Cancer Research Center and Department of Immunology, Semnan University of Medical Sciences, Semnan, Iran; j Department of Physiology, Faculty of Medicine, Infectious Diseases Research Center, Gonabad University of Medical Sciences, Gonabad, Iran; k Guilan Road Trauma Research Center, Trauma Institute, Guilan University of Medical Sciences, Rasht, Iran

**Keywords:** Toll-like receptors. recurrent pregnancy loss (RPL), Therapy, Signaling pathway

## Abstract

Recurrent pregnancy loss (RPL), often known as spontaneous miscarriages occurring two or more times in a row, is a reproductive disease that affects certain couples. The cause of RPL is unknown in many cases, leading to difficulties in therapy and increased psychological suffering in couples. Toll-like receptors (TLR) have been identified as crucial regulators of inflammation in various human tissues. The occurrence of inflammation during parturition indicates that Toll-like receptor activity in tissues related to pregnancy may play a crucial role in the onset and continuation of normal function, as well as in various pregnancy complications like infection-related preterm. TLRs or their signaling molecules may serve as effective therapeutic targets for inhibiting premature activity. At the maternal-fetal interface, TLRs are found in both immune and non-immune cells, such as trophoblasts and decidual cells. TLR expression patterns are influenced by the phases of pregnancy. In this way, translational combinations like epigenetics, have indicated their impact on the TLRs.Importantly, abnormal DNA methylation patterns and histone alterations have an impressive performance in decreasing fertility by influencing gene expression and required molecular and cellular activities which are vital for a normal pregnancy and embryonic process. TLRs, play a central duty in the innate immune system and can regulate epigenetic elements by many different signaling pathways. The potential roles of TLRs in cells, epigenetics factors their ability to identify and react to infections, and their place in the innate immune system will all be covered in this narrative review essay.

## 1 Introduction

In an immunologically distinct location, the maternal-fetal interface must maintain host defense against potential pathogens while fostering tolerance to the allogeneic fetus. Preterm labor, intrauterine growth retardation (IUGR), abortion, and preeclampsia are among the pregnancy issues for which clinical research has shown a high correlation with intrauterine bacterial or viral infections [1]. Thus, the fate of the pregnancy may be significantly impacted by early immune responses to microorganisms at the maternal-fetal interface. The body's first line of defense against invading infections is the innate immune system. It can react fast by identifying the difference between "infectious non-self" and "non-infectious self" [2]. To add insult to injury, the activation of innate immunity is necessary for the creation of acquired immunity that is specific to antigens. At the mother-fetal interface, innate immunity helps to allow the formation of a healthy microenvironment throughout pregnancy, the removal of "infectious non-self" (such as viruses, bacteria, and other pathogens), and tolerance to "non-infectious self" (including the mother, the placenta, and the baby). Increasingly, it appears that the innate immune system is indeed triggered during the perinatal stage. For example, it is well known that innate immune cells, including macrophages, dendritic cells, and natural killer (NK) cells, enter the decidua and cluster around the trophoblasts that are invading the area [3]. Throughout pregnancy, these immune cells not only increase in number but also become more active [4]. Pattern recognition receptors (PRRs) are surface receptors specific to microbes that are generated by cells of the innate immune system. Receptors identify pathogen-associated molecular patterns (PAMPs) and bind to them. Non-immune cells, including epithelial cells, possess PRRs enabling them to detect and react to PAMPs. PAMPs ligate PRRs, which triggers an inflammatory response to the invasive pathogen [5]. Rather than the mannose-binding receptor and scavenger receptor, the majority of this study will concentrate on TLRs, which constitute the primary family of PRRs. We will talk about TLR expression and function at the maternal-fetus interface, as well as how they impact the relationship between the mother's immune system and the trophoblast. Epigenetic processes, such as DNA methylation and histone changes, are essential for controlling gene expression patterns during different phases of reproductive development. Abnormal epigenetic patterns have been associated with infertility, failure of embryo implantation, and RPL. TLRs, crucial elements of the innate immune system, have demonstrated the ability to engage with epigenetic pathways, therefore impacting fundamental cellular processes necessary for healthy conception and embryonic development. Multiple research have investigated the correlation between TLR signaling and epigenetic alterations in the field of reproductive health. TLR activation has been linked to changes in DNA methylation patterns and histone acetylation levels in trophoblast cells. These changes may affect placental development and the immunological tolerance between the mother and fetus. In addition, some environmental variables, such as bacterial or viral infections, can activate TLR-mediated inflammatory reactions, resulting in epigenetic alterations that can contribute to infertility or problems during pregnancy. Epigenetic modifications can impact the activity of genes related to the development of follicles, the ability of the endometrium to receive embryos, and the process of embryo implantation. Recent studies have revealed the possible influence of epigenetic regulation on the modulation of TLR expression and function. Distinct epigenetic markers have been discovered that can either amplify or inhibit TLR signaling, possibly impacting the inflammatory response and reproductive results. Understanding the complex relationship between epigenetics and TLR signaling in infertility and RPL might lead to the creation of new diagnostic tools and tailored treatments. Researchers want to enhance fertility outcomes by discovering precise epigenetic indicators and manipulating TLR activation to restore optimal reproductive function.

## 2 Toll-like receptors

### 2.1 Receptors and their ligands

PAMPs found in bacteria, viruses, fungi, and parasites can be recognized by transmembrane proteins known as TLRs. Leucine-rich repeat motifs seen in these extracellular domains of TLRs have been preserved throughout evolution. To date, eleven mammalian TLRs (TLR1 through TLR11) have been identified; however, human documentation of functional TLR11 proteins has not been found [6]. The specificity of each receptor like exogenous and endogenous ligands is different (Table 1). The host cell requires TLR4 to respond to the lipopolysaccharide (LPS) of gram-negative bacteria [7]. Fungal zymosan, lipoteichoic acid (LTA), gram-positive bacterial peptidoglycan (PDG), and bacterial lipoproteins are all detectable by TLR2, which is the most broadly specific receptor [8]. It appears that TLR2 heterodimerization with other TLRs broadens the spectrum of ligands to which it exhibits a response. Consequently, TLR1/2 heterodimers exhibit a response to a panel of lipoproteins that are separate from those identified by TLR2/6 [9]. It appears that TLRs 3, 7, and 8 are crucial for the body's defense against viruses. It is well recognized that TLR3 binds double-stranded RNA from viruses [10] whereas single-stranded RNA interacts with TLRs 7 and 8 [11]. Cytosine–guanine pairings, or "CpG" patterns, are recognized by TLR9, which enhances cell responses to bacterial DNA. The Herpes virus can also activate TLR9 [12]. TLRs detect molecules created by infections and also interact with other internal substances in the host, usually in response to threats. The danger-associated molecular pattern consists of surfactant protein A, fibrinogen, high-mobility group box protein 1 (HMGB1), and reactive oxygen species (ROS). In addition, the extracellular matrix produces fibronectin fragments, hyaluronic acid oligosaccharides, and eosinophil-derived neurotoxin (EDN) [13,14]. There have been reports indicating that heat-shock proteins (Hsp), including Hsp60, Hsp70, and Hsp90, exhibit interactions with TLRs. However, there exists significant dispute over the specific characteristics of these interactions [15].

**Table 1:** Toll-like receptors and ligands.

Ligand		
TLR	Endogenous	Exogenous
TLR10		
TLR9	Autoimmune chromatin-IgG complex	Non-methylated CpG DNA, Herpes virus
TLR8		Single-stranded RNA
TLR7		Single-stranded RNA
TLR6		Diacylated lipoprotein (with TLR2)
TLR5		Flagellin
TLR4	Proteins Hsp60, Hsp70, and Hsp90, together with ROS and HMGB1, as well as Surfactant protein A, Fibrinogen, Fibronectin, Hyaluronic acid, oligosaccharides, Eosinophil, and derived neurotoxic substances	Lipopolysaccharides, paclitaxel
TLR3	Host RNA	Double-stranded RNA
TLR2		Peptidoglycan, Lipopeptides, Lipoteichoic acids, Zymosan
TLR1		Triacetylated lipoproteins (with TLR2)

### 2.2 TLR signaling

**Fig. 1:**
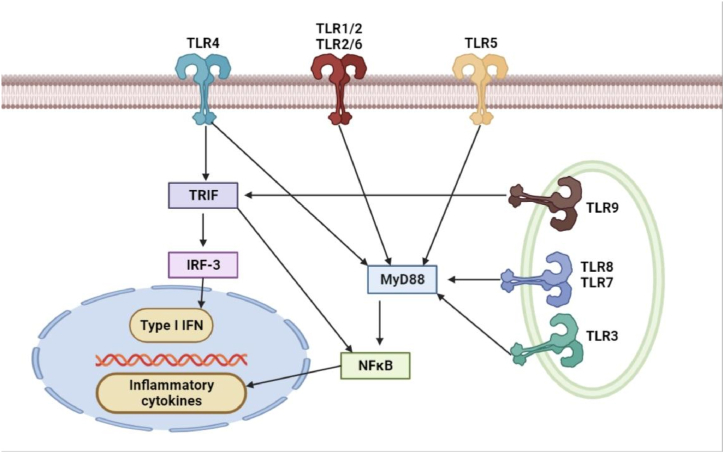
Signals created by the TLR. Membrane TLRs (TLR1, 2, 4, 5, 6) are accountable for the identification of exterior signals, whilst cytoplasmic TLRs (TLR3, 7, 8, 9) are accountable for the recognition of internal signals or signals that originate from within the cell. After ligation, the majority of TLRs trigger the activation of NFjB and the generation of cytokines in a way that is dependent on MyD88. TLR4 is also capable of signaling in a manner that is dependent on MyD88, which is the factor that initiates the production of type I interferons (IFN) and proteins that are induced by IFN. This is similar to how TLR3 accomplishes this. INF, TRIF (Toll-like IL-1 receptor domain-containing adaptor producing IFN-b), and IRF3 (IFN regulatory factor) are essential components of the immune response.

Through a similar intracellular signaling route, TLR binding typically initiates the synthesis of antimicrobial agents and cytokines (Fig. 1). Myeloid differentiation factor 88 (MyD88), an intracellular signaling adaptor protein, is recruited by TLRs upon ligand recognition. When this recruiting process starts, it sets off a chain reaction of kinase events that eventually activate the NFjB pathway, which in turn causes an inflammatory response [16]. Moreover, it has been noted that TLR3 and TLR4 can start signaling pathways without the assistance of MyD88 [17]. Toll/IL-1 receptor domain-containing adaptor-inducing IFN-b (TRIF) is an adapter protein that aids in the process of signaling. TRIF phosphorylates IFN regulatory factor-3 in addition to activating the NFjB pathway (IRF-3). This different route results in the generation of type I interferons (IFNs) and IFN-inducible genes, which are linked to the antiviral response [18].

## 3 Toll-like receptor expression at maternal-fetal interface

### 3.1 Placental tissue

The human placenta shows the expression of all ten TLRs, as well as additional coreceptors and auxiliary proteins including CD14. Mitsunari et al. used reverse transcription polymerase chain reaction (RT-PCR) to show the presence of TLR2, 3, 4, 5, 6, and 9 in cytotrophoblast and syncytiotrophoblast-rich cells obtained from cultured cells separated from term placenta. In the research carried out by Klaffenbach et al. [19], it was shown that the choriocarcinoma cell lines JAR and BeWo express their mRNA for TLR1-10, co-receptors, auxiliary proteins CD14, MyD88, MD-2, TIRP, TRAP, and TRIF. Additionally, the researchers found that these proteins expressed their mRNA. Previous research has shown that TLR1, 2, 3, and 4 are expressed in trophoblast cell lines Swan 71, 3A, and HTR8, as well as in primary trophoblasts that are in their first trimester of development. Both of these cell types do not show any indications of TLR6 expression [20]. The data suggest potential roles of TLR signaling in the placenta during pregnancy. TLR expression in the placenta appears to be controlled both in terms of time and location, rather than remaining constant. For instance, trophoblasts in the third trimester demonstrate the presence of TLR6, but those in the first trimester do not exhibit this characteristic [21]. This suggests that TLR6 expression is regulated throughout time [22]. The term placenta had a greater amount of TLR4 expression compared to the first-trimester placenta upon evaluation. According to the study, compared to term tissue, the placenta in early pregnancy may be less sensitive to pathogen stimulation. However, further investigation is required to elucidate the processes that regulate the modulation of temporal TLR activity. The modulation of TLR activity has spatial characteristics. We discovered that extravillous trophoblast and villous cytotrophoblast, but not syncytiotrophoblasts, express TLR2 and TLR4 in the first-trimester placenta. We hypothesized that placental tissue can only react to a microorganism that has penetrated this outer layer since the syncytiotrophoblast, or the outer trophoblast layer, does not produce TLR. Therefore, a microbe will only become dangerous to the fetus if it manages to penetrate the placental villous or decidual compartments and break through the TLR-negative syncytiotrophoblast layer [22]. There have also been reports of TLR expression in different placental cell types. Immunohistochemical analysis revealed the expression of TLR4 in Hofbauer cells, a specific subset of localized inside the placental villi [23]. Another researcher's most recent study utilized immunohistochemistry to evaluate the expression of TLR2 and TLR4 by macrophages in placentas from the third trimester of pregnancy [24]. While TLR2 expression was more prominent in endothelial cells, macrophages, syncytiotrophoblast, and fibroblast, TLR4 labeling was more evident in these cell types [25]. These findings suggest that trophoblasts and other cell types, in addition to immune cells, can respond to invasive infections and may play a similar role to the innate immune system in the placenta's physiological defense against infection.

### 3.2 Decidua and amnion

There is a lack of understanding regarding the expression of TLR in the decidua in comparison to placental tissue. In two recent research, it was shown that TLR expression may be observed in human decidua. In their study, Krikun and colleagues discovered that mRNA for each of the ten TLRs was detectable in the decidua of the first trimester and the term. Furthermore, they demonstrated that decidual cells from the first trimester express TLRs 2 and 4. By employing immunocytochemistry to investigate the expression of TLR1-6 in primary cultures of decidual cells derived from third-trimester pregnancies, Simhan and Canavan were able to validate the findings. TLR4 was shown to be present on the outer surface of the amniotic epithelium, which indicates that it can detect pathogens in the amniotic fluid, according to the findings of the investigation conducted by Dulay. According to the findings of the study, soluble TLR2 was found in amniotic fluid. This finding means that the capacity of TLR2 to identify TLR2 ligands may be affected by this presence. Based on these findings, it can be deduced that the TLR system plays a role in the regulation of the inflammatory response of the amniotic fluid when it is present in the context of microbial infections.

### 3.3 TLR function at the maternal-fetal interface

Regarding the regulation of immunological responses at both the local and systemic levels during pregnancy, the next inquiry will concentrate on the functions that TLRs play in these cells and how they impact the management of these responses. As a result of the fact that TLRs are widely expressed at the interface between the tissues of the mother and the fetus, which includes both immune and nonimmune cells including trophoblasts, decidual cells, and amniotic epithelium, this is of utmost significance. In the next part, we will discuss the possible roles that TLRs play in the relationship between the mother and the fetus. The activities of TLR2 and TLR4 in the maternal–interface are well recognized because these receptors are the key ones responsible for identifying components of bacterial cell walls. Holmlund et al. gave the first account of the TLR function in the placenta. The study's findings showed that when exposed to zymosan and lipopolysaccharide (LPS), placental cultures in the third trimester produced interleukin-6 (IL-6) and IL-8, which in turn triggered TLR2 and TLR4. This suggests that trophoblasts can identify microbes and trigger immunological responses by stimulating immune cells [26]. Our research shows that the response patterns of first-trimester trophoblasts expressing TLRs vary significantly depending on which TLR is stimulated. For example, when first-trimester trophoblasts have their TLR4 receptors bound to LPS, they show a delayed inflammatory response with just a little rise in cytokines [25]. However, PDG communicates through TLR2 and kills trophoblasts in place of eliciting a cytokine response. The kind of stimulus has an impact on the response pattern following TLR ligation as well. TLR4 activation by Chlamydia heat shock protein 60 may trigger trophoblast death, even if LPS did not in the first trimester [27]. The varying downstream signaling events and distinct ways that various TLR4 ligands employ adaptor molecules might account for the differing effects of these ligands. TLR2 responded differently to the same receptor ligation as well. First-trimester trophoblasts have been shown to undergo apoptosis upon TLR2 ligation by PDG and UV-inactivated human cytomegalovirus (HCMV) [28]. Conversely, Mitsunari et al. found that monophage-activating lipopeptide-2 (MALP-2) from Mycoplasma fementans signaled TLR2 and enhanced the production of prostaglandin E2 and cyclooxygenase (COX)-2 utilizing third-trimester trophoblasts [29]. The existence of TLR6 in third-trimester trophoblasts may account for the variation in efficacy between first- and third-trimester trophoblasts. Based on our discussion, it seems that the reaction that occurs after TLR2 activation requires the cooperation of TLR1 and TLR6 receptors. According to our in vitro research, the pro-apoptotic impact observed following PDG administration is mediated by TLR1 and TLR2 heterodimers. These heterodimers then activate caspase-8, caspase-9, and caspase-3 via the MyD88 FADD pathway. On the other hand, TLR-6 could alter the nature of the reaction by stopping cell death and inducing a cytokine response through NFjB activation [30]. Additionally, it is demonstrated that trophoblast cell migration was reduced by LPS-induced TLR4 ligation [21]. This action might account for the partial trophoblast invasion of the uterine spiral arteries seen in pre-eclamptic individuals.

## 4 The role of TLR3 in TLR signaling-induced antiviral response

**Fig. 2:**
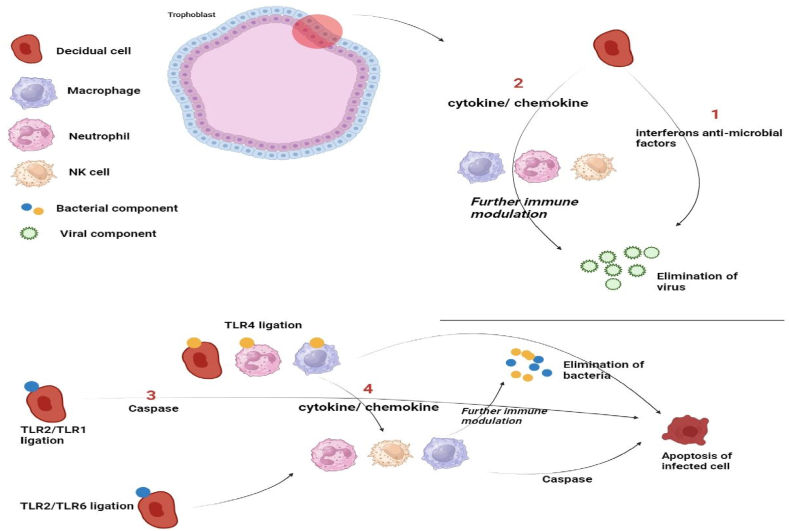
Trophoblasts use TLR3 to identify viral components, and in response, they produce interferons and antimicrobial compounds to inhibit the spread of the virus [1]. Furthermore, trophoblasts generate chemokines and cytokines that influence the mother's immune system in a modulatory manner [2]. TLR2 ⁄ TLR1 ligation in the trophoblast induces death in response to bacterial infection [3], whereas TLR2 ⁄ TLR6 ligation or TLR4 ligation stimulates the trophoblasts' production of cytokines [4]. The trophoblast-triggered inflammatory response stimulates neutrophils, NK cells, and macrophages for further immune regulation.

The fetus may be seriously threatened by viruses that the placenta may be exposed to in addition to bacteria. The trophoblast responds differently to viral infections. First-trimester trophoblasts are known to express TLR3, a receptor that controls the body's immune response to viral dsRNA [31]. Trophozoblasts are stimulated by the synthetic dsRNA PolyA to release antimicrobial and pro-inflammatory cytokines. After treatment with poly, we were able to report the production of interferon-b (IFN-b) utilizing first-trimester trophoblast [31]. The trophoblast may initiate a conventional antiviral response as soon as it detects a virus since IFN-b production is necessary for building an antiviral response. The production of antimicrobial agents by trophoblasts includes the production of interferon and oligoadenylate synthetase (OAS), secretory leukocyte protease inhibitor (SLPI), Myxovirus resistance A (MxA), and apolipoprotein B mRNA-editing enzyme-catalytic polypeptide-like 3G (APOBEC3G). According to these results, the placenta—and trophoblasts in particular—acts as an active barrier to stop some viral infections from infecting the fetus [32]. All of these findings collectively imply that trophoblasts can use TLRs to identify bacterial or viral products and trigger distinct reactions (Fig. 2). The variables related to the kind of reaction have the potential to impact the outcome and are linked to complications during pregnancy, including IUGR, premature labor, and pre-eclampsia.

### 4.1 TLR signaling alters the function of immune cells

Relatively recently, there has been speculation that trophoblast cells may be capable of modulating the immune system by regulating the activities of various immune cells at the interface between the mother and fetus [31]. Prior studies have demonstrated that trophoblasts during the initial trimester could attract monocytes, macrophages, NK cells, and neutrophils. It is indicated that they also exhibit a constitutive secretion of cytokines and chemokines, including GRO-a, MCP-1, and IL8 [33]. After TLR4 or TLR3 agonists ligate trophoblasts, the production of cytokines and chemokines is further enhanced, and the recruitment of immune cells is significantly enhanced [34]. Furthermore, by controlling the differentiation and activation status of the mother immune cells, the substances secreted by trophoblasts exert a strong modulatory influence on them. For instance, macrophages and monocytes cultured with tropho-blasts or their condition medium lose some of their sensitivity to LPS activation [33]. Drawing from these findings, we suggest that the trophoblast has the capacity to "teach" immune cells, with signals from the trophoblast influencing the subsequent actions of the cells. A healthy pregnancy may depend on this appropriate trophoblast-immune cell cross-talk, and alterations or abnormalities in this relationship may cause pregnancy problems.

## 5 Toll-like receptor in pregnancy complications

TLR function has a crucial role in several pregnancy problems including abortion, preterm labor, hypertension, and fetal abnormalities, as shown by animal models and clinical observations. We will first evaluate research conducted with animal models, and then proceed to clinical trials.

### 5.1 Clinical study of TLR role in human pregnancy

TLRs have been associated with pregnancy problems in recent clinical research. In the next part, we will go over the most important findings.

### 5.2 TLR expressions at the maternal-fetal contact during pregnancy

One of the most significant contributing factors to premature birth is recognized to be an intrauterine infection and the chorioamnionitis (CAM) that follows [35]. In chorioamniotic membranes from full-term spontaneous labors and preterm babies linked to chorioamnionitis, we assessed the expression levels of TLR2 and TLR4. Compared to women who were not in labor, women who naturally went into labor at full term had significantly higher TLR2 and TLR4 mRNA levels in their membranes. CAM patients showed significantly higher TLR2 expression in chorioamniotic membranes than non-CAM patients. TLR2 expression in non-CAM preterm labor was limited to the basal surface of amniotic epithelial cells. Positive staining was widely and strongly found throughout the cytoplasm of the epithelium in instances with CAM [25]. On the other hand, trophoblasts from CAM patients expressed less TLR2 than those from non-CAM patients, as demonstrated by Rindsjo et al. These results imply that various areas of the maternal interface have varied responses to infection [36]. But we also need to examine the potential that these differences might be due to differences in study groups' technological setup. Regarding TLR4, Kumazaki et al. [37] demonstrated that preterm placentas with CAM expressed more TLR4 in their villous Hofbauer cells than either term placenta or preterm placenta without CAM. TLR4 was recently shown to be present in the amniotic epithelium. In individuals with chronic myocarditis, TLR4 was most prominently expressed in the basal membrane. The scientists discovered that TLR4 may translocate from the apical to the basal membrane in reaction to an infection. This would reduce TLR signaling at the onset of infection while maintaining the amniotic epithelium's ability to protect against invading microorganisms [22]. This study looked at how cell adhesion molecules (CAMs) and Toll-like receptors (TLRs) contribute to the development of preeclampsia. The TLR4 expression in trophoblast cells was notably elevated in women who had preterm birth due to preeclampsia compared to those who had preterm delivery with or without complementary and alternative medicine (CAM) therapies. Moreover, it was demonstrated that the expression of TLR4 exhibited co-localization with activated NFjB, Tumour necrosis factor-alpha (TNF-α), and M30, a marker unique to apoptosis in epithelial cells. The observations suggest a potential relationship between inflammatory cytokines and the activation of TLR4 expression, leading to an enhanced trophoblast responsiveness to TLR ligands [38]. An association between elevated TLR4 expression in microvessel endothelial cells isolated from placental villi and defective umbilical artery Doppler studies was established. These results imply that certain pathogens or endogenous chemicals produced during inflammation control the level of TLR expression in the placenta, while the precise mechanisms underpinning these findings are yet unknown. This regulation serves as a feedback loop to either stimulate or block additional immune responses. The capacity of TLRs to identify host products, also referred to as "danger signals," which are released by damaged cells, in addition to microbial ligands, is a novel aspect of their function [39], suggesting that infections and non-infection-associated disorders connected to pregnancy may be mediated by TLRs. As an illustration, Holmlund et al. [40] showed that decidua from preeclamptic individuals have elevated levels of HMGB1, a ligand for TLR4. It has also been demonstrated that antiphospholipid antibodies, which are implicated in the pathogenesis of recurrent miscarriages, preeclampsia, and premature labour, increase the inflammatory capacity of first-trimester trophoblasts via the TLR4 pathway [41].

### 5.3 TLR polymorphisms and pregnancy

**Fig. 3:**
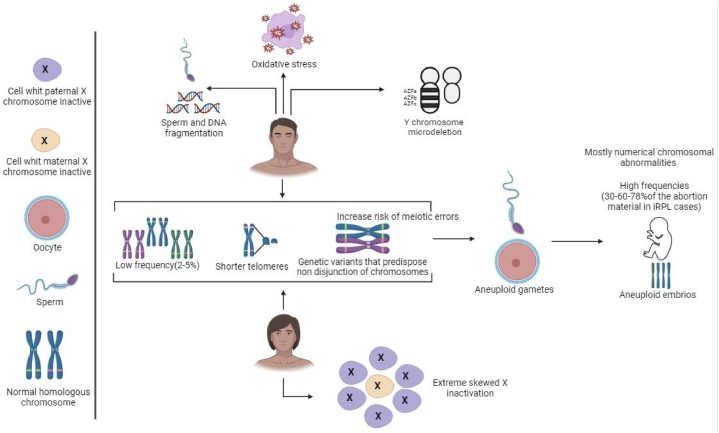
Role of different translational factors alongside the direct influence of oxidative stress in the reproduction process.

TLR polymorphisms may have an impact on a person's vulnerability to pregnancy disorders, as the TLR system is implicated in several reproductive diseases. Much research examined the possibility of a link between pregnancy problems and TLR polymorphisms and different genes (Table 2). The majority of research on preterm labor focuses on polymorphism in TLR2 and TLR4. It's interesting to note that studies have linked fetal polymorphism and preterm labor susceptibility not only to the mother's polymorphism but also to the infant's. These findings imply that the placenta or fetus's immune system also participates in regulating the mother's innate immunological reaction to prevent adverse outcomes during pregnancy [39]. Babies with two polymorphic TLR2 alleles had significantly shorter gestational ages, according to a study that examined the genomic DNA of neonates [42]. A separate study conducted on the Finnish population has revealed an association between the presence of Gly in TLR4 299 and the occurrence of preterm labor in both maternal and neonatal populations. A study in Uruguay also noted this similar tendency [43]. TLR4 polymorphisms are also linked to premature delivery, which is a documented side effect of bacterial vaginosis (BV). According to one study, women with BV had considerably lower Thr for TLR4 than those in the absence of BV [44]. Another study linked higher levels of anaerobic gram-negative rods, vaginal pH, and Gardnerella vaginalis to gly for TLR4, which is known to reduce LPS responses. This finding contradicts the findings of the first study [45]. Additionally, polymorphisms in TLRs affect the chance of developing preeclampsia. A recent study conducted by van Rijn et al. suggests that maternal TLR4 polymorphisms may affect a person's susceptibility to high liver enzymes and low platelets (HELLP) syndrome, early-onset preeclampsia, and other diseases [46]. Furthermore, Hirschfeld and colleagues observed that two SNPs in the TLR 4 gene as well as an SNP in the TLR 2 gene exhibited associations with normal pregnancy controls. Furthermore, Hirschfeld and colleagues observed that two single SNPs in the TLR 4 gene, as well as an SNP in the TLR 2 gene, exhibited associations with normal pregnancy controls [45]. The particular mechanism behind each sickness requires further investigation, but these clinical data indicate that the TLR systems are important in pregnancy issues and the role of different translational factors alongside the direct influence of oxidative stress are of great importance (Fig. 3).

**Table 2:** Most important and associated genes with different details include rs code, common names, and also biological effects.

Gene	Common names	rs code	Biological effect	Reference
F2	G20210A	rs1799963	Thrombophilia	[47]
MIR449b	Unspecified	rs10061133	Epigenetic	[48]
MMP9	−1562C/T	rs34016235	Remodeling of extracellular matrix endometrium	[49]
VEGFA	−2549 I/D	rs35569394	Vascular function	[50]
MMP2	−735C/T	rs2285053	Remodeling of extracellular matrix endometrium	[49]
MTHFR	C677T	rs1801133	Thrombophilia	[51]
VEGFA	−1154G > A	rs1570360	Vascular function	[52]
PAI-1	4G/5G	rs1799889	Thrombophilia	[53]
PAI-1	−844G > A	rs2227631	Thrombophilia	[54]
PAI-1	11053T > G	rs7242	Thrombophilia	[54]
PGR	G/T - Val660Leu (PROGIN)	rs1042838	Oocyte maturation, implantation, and maintenance of the placenta	[55]
IL-10	2195 A > G	rs1518111	Immune tolerance	[56]
IL-6	−634C/G	rs1800796	Immune tolerance	[56]
IL-1β	−511T > C	rs16944	Immune tolerance	[57]
IL-17	G-197A	rs2275913	Immune tolerance	[58]
IL-18	137G/C	rs187238	Immune tolerance	[59]
IL-10	−819C/T	rs1800871	Immune tolerance	[60]
F13A1	Y205F-A614T	rs3024477	Thrombophilia	[61]
SELP	C-2123G or N562D	rs6127	Immune tolerance	[62]
MTHFR	A1298C	rs1801131	Thrombophilia	[63]
F5	factor V Leiden	rs6025	Thrombophilia	[64]
TP53	p53 Arg72Pro or p53 codon 72	rs1042522	Vascular function and embryo development	[65]
THBD	C1418T	rs1042579	Thrombophilia	[66]
EPCR	1652C/G	rs867186	Thrombophilia	[67]
RAN	Unspecified	rs14035	Epigenetic	[68]
TNF-α	−863C > A	rs1800630	Immune tolerance	[69]
MIR423	Unspecified	rs6505162	Epigenetic	[70]
XPO5	Unspecified	rs11077	Epigenetic	[68]
MIR125a	Unspecified	rs12976445	Epigenetic	[71]
SERPINC1	786G > A	rs2227589	Thrombophilia	[72]
F13A1	C1694T or Pro564Leu	rs5982	Thrombophilia	[61]
PKR2	Unspecified	rs6053283	Vascular function	[73]
VEGFR-2	1719A/T	rs1870377	Vascular function	[74]
eNOS	G894T	rs1799983	Vascular relaxation contraction	[59]
F13A1	Val34Leu-G103T	rs5985	Thrombophilia	[75]
FOXP4	−3279C/A	rs3761548	Immune tolerance	[69]
FOXP5	del/ATT	rs5902434	Immune tolerance	[69]
FOXP6	Unspecified	rs2294021	Immune tolerance	[69]
DICER	Unspecified	rs3742330	Epigenetic	[69]
MIR27a	Unspecified	rs895819	Epigenetic	[48]
TGF-β1	G915C or Arg25Pro	rs1800471	Immune tolerance	[76]
CTLA-4	+49A/G	rs232775	Immune tolerance	[56]
DROSHA	Unspecified	rs10719	Epigenetic	[69]
MIR125a	Unspecified	rs41275794	Epigenetic	[71]
FOXP3	−924 A/G	rs2232365	Immune tolerance	[69]
ACE	I/D	rs1799752	Thrombophilia	[77]

Antigens that are self-antigens and those that are not self-antigens are normally distinguishable by the immune system. Tregs are also known as regulatory T cells. Tolerating the relationship between the mother and the fetus during pregnancy is an essential function of Tregs [78]. Some data suggest an association between a decrease in CD4^+^ CD25^+^ Tregs and the prevalence of sexually transmitted infections (RSA), with 79 instances being documented. The phenomena that were observed can be related to the downregulation of FOXP3, which is an essential regulator that plays a role in the creation and functioning of CD4^+^ CD25^+^ Tregs through the NFAT pathway [79]. A subset of individuals in the Iranian RSA research has a much higher frequency of −924A/G and −20G/A single nucleotide polymorphisms (SNPs) inside the FOXP3 gene, as indicated by the findings gained from this analysis. Even though the 924A/G variant is located inside the binding domain of GATA-3, the A allele is necessary for the promoter to bind. Therefore, the inhibition of the Th2 immune response is the cause of the link that has been shown between the G allele, the G/G genotype, and the occurrence of abortion-related events [80]. Treg cells use CTLA-4, a negative regulator of T cells, to create endometrial tolerance to the fetus to facilitate successful implantation [81,82]. The cytokine IL-6 also impacts the balance of Th17 and Treg cells by either inducing or inhibiting the development of Th17 and Treg cells, respectively [83]. Based on existing studies, a subgroup of Iranian patients with the G allele in the CTLA-4+49A/G polymorphism is protected against RPL. Moreover, a substantial correlation was found between RPL and the IL-6 634C/G polymorphism, where the G allele was associated with a five-fold increase in RPL occurrence. Consequently, the scientists deduced that the IL-6 variation, 634C/G, and the CTLA-4 single SNP, +49A/G, would be risk factors for RPL in Iranian women [84]. A different study also found that among Iranian patients, the +49 G allele reduced the chance of RSA [56]. GITR and CTLA-4 are two of the markers that identify three cells [85]. Another cytokine associated with Treg cells that control IFN-g and TNF is IL-10 [82]. The study observed a cohort of Iranian patients with rheumatoid arthritis and discovered a significant decrease in the expression levels of CTLA-4 and GITR when compared to a control group. Furthermore, they noticed that RSA individuals had higher IL-10 expression than control participants [86]. Treg and Th17 cells are produced from CD4+T cells, and these cells are differentiated by TGF-β and IL-6. The presence of transforming TGF-β and IL-6 triggers the formation of Th17 cells, whereas TGF-β promotes the differentiation of Treg cells. Pregnancy loss has been linked to decreased levels of IL-6 and increased levels of transforming TGF-β [87]. It has been demonstrated that compared to normal nonpregnant people, a subset of Iranian patients with RPL had significantly greater expressions of IL-6, IL-23, and IL-17. In addition, the RPL instances' TGF-β and FOXP3 expressions were lower than those of typical non-pregnant individuals [88]. Th17 cells, which are similar to Treg cells, release IL-17, which controls the immune system's rejection of foreign tissues [89]. Thus, pregnancy loss may be caused by an imbalance between Th17 and Treg [90]. When compared to normal nonpregnant individuals, a subgroup of Iranian URSA patients was shown to have considerably higher blood levels of Th17-related cytokines, such as IL-17, IL-21, and IL-22. However, they discovered that compared to controls, URSA had much-reduced amounts of the cytokine TGF-β associated with T cells [91]. Co-stimulatory and inhibitive signals also govern immunity mediated by cells [92]. The primary regulator of Treg cells, known as FOXP3, experiences a decrease in expression due to the action of OX40, a cell surface costimulatory protein that is generated by activated CD4 and CD8 T cells. OX40 also controls the PI3K/Akt, calcium/NFAT, and NF-κB signaling pathways [93]. It was discovered that a subgroup of Iranian RSA cases displayed higher levels of OX40 and OX40L in comparison to healthy individuals. One finding that was made on RSA risk factors was elevated blood OX40L levels [94]. Thus, the genetic variables linked to each patient are completely unique (Table 3) [95].

**Table 3:** Every genetic component linked to patients' repeated miscarriages.

Gene	Population	Results	Reference
SYCP3	100 cases	RPL risk was connected with polymorphism	[96]
	100 controls		
VEGF	50 cases	RPL risk and polymorphism were associated	[97]
	50 controls		
CAT	105 cases	Polymorphism was linked to RPL risk	[98]
	90 controls		
IL−17	85 cases	The polymorphism was linked to the risk of RPL	[99]
	85 controls		
IL−18, IL−33	300 cases	The risk of RPL was linked to polymorphism	[100]
	300 controls		
VEGF	10 cases	Higher serum levels	[101]
TNF‐α	65 cases	Polymorphism was correlated with RPL risk	[102]
	65 controls		
CTLA−4, GITR, IL−10	20 cases	CTLA−4 and GITR underexpression	[86]
	20 controls	IL−10 overexpression	
IL−10	139 cases	A link was found between polymorphism and RPL risk	[103]
	143 controls		
IL−17, IL−21, IL−22, TGF‐β	46 cases	Higher serum levels of IL−17, IL−21, and IL−22	[91]
	28 controls	Lower serum levels of TGF‐β	
IL−6, IL−23, IL−17, FOXP3, TGF‐β	20 cases	IL−6, IL−23, and IL−17 overexpression	[88]
	20 controls	FOXP3 and TGF‐β underexpression	
SLC19A1	147 cases	The risk of RPL was associated with polymorphism	[95]
	150 controls		
OX40	40 cases	Overexpression	[94]
	40 controls		
CTLA−4	120 cases	Polymorphism was correlated with RPL risk	[104]
	120 controls		
IL−10	85 cases	Polymorphism was correlated with RPL risk	[105]
	104 controls		
IL−6	8 cases	IL−6 underexpression following vitamin D treatment	[106]
	8 controls		
IL−6 and CTLA−4	120 cases	Multiple genetic differences were linked to a higher risk of RPL	[56]
	120 controls		
SYCP3	100 cases	Interactions between polymorphism and RPL risk	[96]
	100 controls		
FOXP3	195 cases	Polymorphism was correlated with RPL risk	[80]
	101 controls		
G‐CSF	122 cases	Polymorphism was correlated with RPL risk	[80]
	140 controls		
BMP4	70 cases	Associations between polymorphism and RPL risk were found	[107]
	100 controls		
USP26	72 cases	Mutation	[108]
miR−21, PTEN	25 cases	miR−21 underexpression	[109]
	25 control	PTEN overexpression	
eNOS	130 cases	RPL risk was linked to polymorphism	[110]
	110 controls		
MTHFR	330 cases	Polymorphism increased RPL risk	[111]
	350 controls		
HLA‐G	117 cases	Polymorphism was correlated with RPL risk	[112]
	117 controls		
ACE	100 cases	Variation and RPL risk were linked	[113]
	100 controls		
SULF1	100 cases	RPL risk associated with polymorphism	[114]
	100 controls		
PAI−1	63 cases	Polymorphism was correlated with RPL risk	[115]
	114 controls		
CD46	141 cases	Polymorphism was correlated with RPL risk	[116]
	153 controls		
MTHFR	100 cases	RPL risk was linked to polymorphism	[117]
	100 controls		
PAI−1	595 cases	Polymorphism was correlated with RPL risk	[118]
	100 controls		
HLA‐G	200 cases	Polymorphism was correlated with RPL risk	[112]
	200 controls		
APOE	81 cases	Polymorphism was correlated with RPL risk	[119]
	81 controls		
Leptin	81 cases	Higher levels of serum leptin	[120]
TNF‐α, TNF‐β, and IL−2	92 cases	Higher serum levels	[121]
	40 controls		
HLA‐G	93 cases	Polymorphism was correlated with RPL risk	[97]
	93 controls		
HLA‐G1	30 cases	Underexpression	[122]
	30 controls		
APOE	100 cases	Polymorphism was correlated with RPL risk	[123]
	100 controls		
CD69 and CD161	43 cases	CD69 and CD161 overexpressions	[124]
	43 controls		
HLA‐E	200 cases	Polymorphism was correlated with RPL risk	[125]
AR	85 cases	Polymorphism was correlated with RPL risk	[126]
	85 controls		
PAI−1	100 cases	Polymorphism was correlated with RPL risk	[53]
	100 controls		
PAI−1	100 cases	Polymorphism was correlated with RPL risk	[117]
	100 controls		
HPA−1	110 cases	Polymorphism was correlated with RPL risk	[109]
	110 controls		
ND1	33 cases	Polymorphism was correlated with RPL risk	[127]
	100 controls		
P53	167 cases	Polymorphism was correlated with RPL risk	[128]
	32 controls		

### 5.4 T-helper cytokines

T helper cells, also known as CD4^+^ cells, are the primary cells responsible for adaptive immune responses. It is T helper cells that give birth to the Th1 and Th2 subtypes. The humoral and cell-mediated immune responses, respectively, are related to the Th1 and Th2 immune responses. Even though cytokines linked to Th2 are associated with healthy pregnancies, pregnancy has been shown to reduce the production of Th1 [129]. Responses that are classified as Th1 include TNF, IFN, IL2, and IL12, whereas responses that are classified as Th2 include IL5, IL6, IL4, and IL10 [130]. Iranian RSA patients exhibited significantly elevated levels of TNF-α, TNF-β, and IL-2 in their serum compared to the control group, indicating the possibility of a connection between Th1 cytokines and the occurrence of miscarriages [131]. In a sample of Iranian patients, a separate group has discovered that there are high connections between RPL and the TNF‐α mutations −863C/A and −238G/A. In addition to the 308G gene, there was another protective factor that prevented spontaneous abortions. The production of cytokines by Th1 and Th2 cells must be in equilibrium for a pregnancy to be considered healthy [132]. Pregnancy termination and increased levels of pro-inflammatory cytokines are directly correlated. However, it is worth noting that IL-10, an anti-inflammatory cytokine, plays a role in inhibiting Th1-mediated cellular mechanisms, which are of utmost importance in maintaining the viability of pregnancy [133]. Th1-mediated cellular responses are inhibited by IL-10 through the suppression of interferon-gamma (IFN-γ) and tumor necrosis factor (TNF) cytokine production. According to the study, compared to the control group, those with RM had a considerably greater frequency of the IL-10 –592 A/C genotype. Moreover, a sample of Iranian participants showed a link between the RM and IL-10-819C/T polymorphism [105]. The study discovered that compared to a control group of healthy individuals, a particular group of Iranian participants who had been diagnosed with RPL had a higher prevalence of the IL-10 -592 CC genotype. This was discovered concerning the genotype's prevalence in healthy people. The results of the investigation showed that those with the 592 CC genotype secreted less IL-10 from their bodies [100]. IL-18 is secreted by a range of immunological and nonimmune cells that control the development of Th1 and Th2 cells [134]. Endothelial cells produce IL-33, which is associated with Th2 activation. A substantial correlation has been confirmed between the IL-18 (rs1946518) polymorphism and RPL. Due to this connection, a certain group of Iranian individuals with the CC genotype may have RPL. Iranian individuals with the GA genotype of the IL-33 polymorphism were shown to have a higher risk of RPL [100]. Th-17 cells use IL-23 to induce the production of IL-17. The frequency of the IL-17F (rs763780) gene polymorphism was found to differ significantly between an Iranian RPL patient cohort and a control group. This result suggests that a high incidence of RPL in this group [99] may be associated with this polymorphism. Granulocyte colony-stimulating factor, or G-CSF for short, is a glycoprotein that is present in both endothelial cells and macrophages. Its OMIM number is 138970. It has been linked to an increase in IL-4 and IL-10 levels, which are anti-inflammatory cytokines. Furthermore, it contributes to tipping the scales in favor of the Th2 reactions over the Th1 reactions [135]. Research that is currently available indicates that there is a substantial difference in the frequencies of the CT and T allele (TT + CT) genotypes of the rs1042658 gene variation between a control group and a sample of Iranian patients with retinopathy of prematurity (RPL). The idea that polymorphism contributes to the development of RPL in Iranians is supported by this [136]. BMP4, a ligand belonging to the TGFβ family, activates the SMAD transcription factors and might affect the regulation of the development of early ovarian follicles [56]. Moreover, conventional BMP signaling promotes CD4 T cell activation. In comparison to the controls, who also had the BMP4 (rs121912765) polymorphism as an RSA risk factor in Iran, it was demonstrated that the Iranian patients exhibited a greater frequency of this polymorphism. This was determined by comparing a sample of Iranian RSA patients to controls who also had this polymorphism [107]. The balance between antioxidants and oxidants is essential for maintaining healthy physiological conditions throughout a successful pregnancy. Because there are insufficient antioxidants during pregnancy, problems connected to ROS arise [137]. ROS is a key player in the regulation of T-cell activity. Lower ROS levels have been shown to drive Th-1 and Th-17 differentiation, whereas higher ROS levels have been seen to improve Th-2-mediated immune responses [138]. By converting hydrogen peroxide into oxygen and water, the vital antioxidant enzyme catalase shields cells from harm caused by ROS [139]. A subset of Iranian cases showed a strong connection between higher vulnerability to spontaneous abortion and the CAT 262C/C genotype [98]. A member of the deubiquitinating enzymes (DUB) family, ubiquitin-specific protease 26 controls the proliferation, differentiation, and carcinogenesis of cells [140]. Stabilizing SMAD7, which in turn modulates TGF- TGF-signalling, is the role of USP26. In addition, TGF- has a variety of impacts, including pleiotropic effects, on the regulation of CD4+T-cell responses and adaptive immunity [141]. It has been shown through research that in a subgroup of Iranian males and females, respectively, USP26 gene variants might be linked to RPL and infertility [108]. The modulation of immunological responses is greatly aided by T-helper (Th) cytokines, whose dysfunction has been linked to infertility. For example, the functions of Th1 and Th2 cytokines differ in reproductive immunity. Overproduction of Th1 cytokines, such as TNF-α and IFN-γ, is linked to inflammatory reactions that are harmful to the maintenance of pregnancy and implantation. On the other hand, Th2 cytokines, such as IL-10 and IL-4, provide anti-inflammatory environments that are favorable for a healthy pregnancy. Reproductive organs produce TLRs, important components of innate immunity that affect Th cytokine production. The Th1/Th2 balance may be thrown off if TLR activation occurs, which might have negative effects on reproduction. According to studies, activation of TLR2 and TLR4 might increase the production of Th1 cytokines, which can exacerbate inflammation and lead to infertility. Comprehending the complex interaction between TLR signaling pathways and T-helper cytokines is essential for formulating effective treatment plans for immune-mediated infertility. This junction of TLR activation and cytokine profiles is a good place to look for future study in reproductive immunology.

### 5.5 Angiogenesis

One essential physiological mechanism for a healthy pregnancy is angiogenesis. VEGF is an angiogenic cytokine that regulates endothelial cell proliferation and differentiation in addition to increasing vascular permeability [142]. The upregulation of VEGF expression can be induced by several stimuli, including hypoxia, EGF, TGF-β, and IL-1β. Previous studies have demonstrated that a certain subgroup of individuals with Iranian undifferentiated rheumatoid seronegative arthritis (URSA) exhibited significantly increased levels of VEGF in their blood [101]. Moreover, another research team has reported that the 18-base pair insertion/deletion polymorphism in the VEGF gene significantly increases the susceptibility to RSA in a cohort of patients from southeastern Iran [97]. A subclass of noncoding RNAs known as micro-RNAs regulates posttranscriptional processes by either blocking translation or causing mRNA degradation [143]. They are vital in the etiology of several illnesses that affect the reproductive system, including RM and preeclampsia [4]. One of the factors linked to miscarriage is aberrant angiogenesis [144]. The miR-21 protein targets PTEN to regulate angiogenesis. Furthermore, increased angiogenesis and elevated VEGF expression are the results of the activation of the AKT and ERK signaling cascades caused by the overexpression of miR-21 [145]. A subset of Iranian RM patients has been shown to have PTEN overexpression and miR‐21 underexpression [146]. Numerous elements of pregnancy, including fetomaternal angiogenesis and blood circulation, are regulated by nitric oxide (NO), which is necessary for a healthy pregnancy [147]. As a result, pregnancy loss and abnormal placental perfusion may arise from decreased NO production [52]. According to Shin et al. (2010), nitric oxide synthases (NOSs) are in charge of producing soluble NO from l-arginine. A subgroup of Iranian RPL patients was shown to have significantly greater frequencies of eNOS −786 T > C variations and eNOS −786C alleles as compared to healthy participants. The eNOS −786C allele was found to enhance the probability of early pregnancy loss [110]. Reduced vascular development has been linked to early pregnancy loss because adequate fetal placental circulation is necessary for a viable pregnancy. In the early trimester of pregnancy, the placenta usually has a modest rate of apoptosis, but as gestation progresses, the ratio increases [148]. The multifunctional transcription factor P53 controls both angiogenesis and cell death [149]. The P53 codon 72 gene polymorphism and RPL were shown to be significantly correlated in research including a sample of Iranian individuals. In particular, participants with the Pro/Pro genotype showed a greater vulnerability to RPL in comparison to participants with the Arg/Arg genotype [150]. The enzyme that breaks down the sulfate ester linkages in heparin sulfates is called arylendosulfatase, or SULF. By removing 6-O-sulfate groups from heparin sulfates, this enzymatic activity modifies the growth factor binding sites found in proteoglycans. Consequently, SULFs and the processes of angiogenesis and embryogenesis could be related. Researchers have shown that in a subgroup of Iranian patients with greater frequencies of homozygous GG and AA genotypes, SULF1 polymorphism is associated with an increased risk of recurrent miscarriage. Additionally, a correlation was seen between the AG genotype with an increased probability of achieving a successful pregnancy, as evidenced by the elevated prevalence of this genotype among individuals who were in good health [151]. The bioenergetic centers of cells, mitochondria play a crucial role in cell growth and division by producing ATP and oxidative phosphorylation [152]. This organelle regulates angiogenesis as a cellular oxygen sensor by using the migration and proliferation of epithelial cells. The NADH dehydrogenase I is a component of the largest complex in the electron transport chain, the NADH dehydrogenase complex. The T4216C variation of ND1, which may be considered a polymorphism with secondary effects on RPL, is present in 30 % of Iranian RPL patients [127]. The process of angiogenesis, or the growth of new blood vessels, is essential for placental development and successful implantation. Infertility and dysregulation of angiogenesis have been related, especially in endometriosis and polycystic ovarian syndrome (PCOS). Important angiogenic factors that are necessary for endometrial receptivity and embryo implantation include VEGF (vascular endothelial growth factor). Inadequate endometrial vascularization caused by impaired angiogenesis can lead to unsuccessful implantation and early pregnancy loss. Reproductive organs express TLRs, which are microbial component detectors that can impact angiogenesis. It has been demonstrated that the activation of TLRs, specifically TLR2 and TLR4, affects the angiogenic factor synthesis. TLR signaling can interfere with normal angiogenesis and increase infertility by upregulating inflammatory pathways and downregulating VEGF production. In order to improve the reproductive outcomes for infertile women, it is imperative to comprehend the interaction between angiogenesis and TLR-mediated immune responses.

### 5.6 Human leukocyte antigens

Major Histocompatibility Complex (HHS) proteins are encoded by human leukocyte antigens (HLAs), which function as immune system regulators. The immune system's ability to distinguish between self and nonself cells is aided by the HLA system. A healthy pregnancy may be linked to HLA expression at the fetomaternal interface [153]. Human leukocyte antigen-G expression has been seen in several anatomical sites, including embryonic trophoblasts, endothelial precursors, and pancreatic islets [154]. Fetal trophoblast cells are shielded from the mother's uterus's NK cells by the HLA-G protein during gestation [155]. RSA susceptibility has been shown to have a significant correlation with HLA-G 3142G > C and 14-bp ins/del polymorphisms in a sample of Iranian patients [156]. This was proved by the data collected from the patients. A connection has been found between the deletion/insertion polymorphism in HLA-G, which is 14 base pairs long, and the control of HLA-expression G. It was shown that a group of Iranian women who had experienced repeated miscarriages had a higher prevalence of heterozygote +14bp [157], in contrast to people who were fertile and served as controls. HLA-G1 expression was significantly lower in the situations where an abortion was threatened than in the control group. HLA-G1 and HLA-G5 expression were associated, and NK cell counts and these cytokines were favorably connected with IL-10 levels. This was in addition to the previously demonstrated correlation between NK cell counts, these cytokines, and IL-10 concentrations. Based on these findings, sustaining the fetus throughout pregnancy requires uterine NK, HLA-G1, and HLAG5 [122]. Another HLA protein that has been connected to the development of fetal-maternal tolerance is HLA-E. It interacts with the CD94/NK G2A complex, an essential component in the NK cell suppression process. The results of the study showed that in comparison to the control group, a subset of patients with rheumatoid arthritis had a higher prevalence of HLA-E 0101 polymorphism. In contrast, there was an increased frequency of HLA-E 0103 in the control group. Furthermore, the maintenance of fetal well-being was found to be associated with the HLA-E0101/0103 heterozygous genotype in the Iranian population [158]. During pregnancy, human leukocyte antigens, or HLAs, are essential for immunological tolerance and recognition. Recurrent pregnancy loss and infertility have been associated with polymorphisms or abnormal expression of HLA genes. To modulate maternal-fetal immunological tolerance, HLA-G in particular is essential. A miscarriage or unsuccessful implantation may arise from an immune-mediated assault on the embryo caused by low HLA-G expression. HLA-C alleles also affect placental development and trophoblast invasion; some alleles are linked to worse pregnancy outcomes. TLRs, which are well-known for their function in innate immunity, can modify immune responses through their interaction with HLA molecules. TLR activation affects the immunological milieu of the reproductive tract by influencing HLA expression. Increased generation of pro-inflammatory cytokines as a result of TLR signaling has the potential to further impair HLA-mediated immunological tolerance mechanisms. Investigating the connection between TLR activation and HLA expression identifies targets for therapeutic intervention and sheds information on possible immunological reasons for infertility.

### 5.7 Natural killer cells

Neurotoxic lymphocytes, also known as NK cells, are linked to the mother's immune system suppression. They are the immune cells that are most common in the uterine implantation site, and they are responsible for providing the first line of defense protection against infections. There are two types of NK cells: CD16^−^CD56bright and CD16^+^CD56dim [159]. Following in vitro fertilization (IVF), an increase in peripheral blood NK cells is linked to a higher chance of anomalous implantation. The contact between NK cells and target cells is regulated by the cell adhesion protein CD56 [160]. Abortion and damage to trophoblast cells are caused by elevated NK cell activity. Research has demonstrated that a subset of RSA patients exhibited significantly higher levels of NK cytotoxicity as compared to controls. Furthermore, compared to control cases, the RSA patients showed a noticeably larger percentage of CD56dim cells [161]. IVF failure and RSA may be related to immunological deficiencies that occur during the interactions between the fetus and the mother's immune cells. Placental damage and increased NK cells are linked, and NK cells may be involved in this immunological interaction [162]. A correlation has been observed between cytokine production and cytotoxicity, as well as the cell surface markers CD69 and CD161 [163]. When compared to healthy people, a sample of cases with RAS and IVF failure showed noticeably higher levels of CD69 NK cells. Additionally, they found that, in contrast to typical cases of successful pregnancy, RSA and IVF failure cases had higher levels of CD161 expression on NK cells. As a result, Iranians' increased expression of CD69 and CD161 on NK cells may be considered a risk factor for RSA and IVF failure [164]. An essential part of the innate immune system, natural killer (NK) cells are important in the early stages of pregnancy. Recurrent miscarriages and infertility have been linked to aberrant NK cell function. Placental development and successful implantation depend heavily on uterine NK (uNK) cells. The maintenance of pregnancy and the implantation of embryos can be adversely affected by an unfavorable immunological milieu caused by an imbalance in NK cell subpopulations or by an overabundance of cytotoxic NK cells. NK cells express TLRs, which can modify the activity of the cells by detecting pathogen-associated chemical patterns. NK cells' TLR activation can improve their cytotoxic capabilities and have an impact on the synthesis of cytokines like IFN-γ. TLR signaling on NK cells may cause implantation failure and miscarriage by altering the sensitive immunological balance needed for pregnancy. Understanding immune-mediated infertility and creating tailored therapeutics need research into the interplay between TLRs and NK cells in the reproductive tract.

### 5.8 Clinical perspective

We have just started researching inflammation at the maternal-fetal interface, with a special focus on TLR-driven processes. A deeper comprehension of receptors and the signal transduction cascades they trigger will help clarify why PTL and premature prelabour rupture of membranes (PPROM) affect certain pregnancies but not others. Furthermore, research on the internal triggers of TLRs might provide insight into the mechanisms behind preterm labor (PTL) and preterm premature rupture of membranes (PPROM) in the absence of infection. According to histological and in vitro research, the key to preventing preterm labor would be to decrease inflammatory reactions brought on by microbial products early on, before positive feed-forward cascades are irreversibly started and spread. Given that TLRs are important upstream regulators of the proinflammatory cascade that leads to preterm labor, it would make sense to investigate these receptors or the signaling molecules they are linked with as potential targets for treatments. Researchers are examining TLR agonists, antagonists, adaptor molecules, and signaling intermediates to find therapeutic approaches for a variety of illnesses, such as autoimmune disorders, asthma, and septic shock [165]. However, since several TLRs could be implicated in the immune response to an infectious disease, targeting adaptor proteins or intracellular signaling molecules common components of various pathways might be the best line of action. component of transcription An increasing amount of evidence indicates that functions as a focal point for inflammatory mediators including TNF-a, LPS, and other TLRs, and that this involvement is important in the pathophysiology and physiology of labor [166]. NFjB is therefore an excellent potential target for PTL treatment and prevention. Currently, studies are being done in vitro to investigate the possibility of ablating relevant labor-promoting pathways by NFjB activity inhibition [167]. It is essential to comprehend the immunological basis of infertility from a clinical standpoint to enhance diagnostic and treatment methods. TLRs are becoming recognized as important contributors to the field of reproductive immunology. Abnormal TLR signaling can result in uncontrolled immunological responses, which are associated with many causes of infertility such as endometriosis, polycystic ovarian syndrome (PCOS), and unexplained infertility. From a clinical perspective, individuals experiencing infertility commonly have increased levels of pro-inflammatory cytokines, which is a result of the activation of TLRs. TLR polymorphisms have been linked to modified immunological responses and negative reproductive effects. Evaluating the expression and functionality of TLR can assist in identifying individuals who are susceptible to immune-related infertility. Furthermore, the therapeutic manipulation of TLR pathways has promise for restoring immunological equilibrium and enhancing fertility results. By using TLR-related diagnostic methods and therapies, medical practitioners may more effectively customize interventions for individuals with infertility, therefore increasing the chances of achieving successful pregnancies.

## 6 Epigenetics and male infertility

### 6.1 The significance of epigenetic mechanisms in spermatogenesis

The ability of male fertilization and sperm function are both critically dependent on epigenetic changes. Sperm production requires correct management of epigenetic processes such as DNA methylation, chromatin remodeling, histone tail modifications, and non-coding RNAs throughout gonadal development and spermatogenesis [168].

### 6.2 DNA methylation

In differentially methylated regions (DMRs), DNA methyltransferases (DNMTs) transfer a methyl group (-CH3) from S-adenosyl methionine to the fifth carbon of the cytosine ring (5meC). The majority of developmental and housekeeping genes are found to possess DMRs inside their promoter regions. For instance, DMRs are frequently observed within the regulatory regions of several genes that exhibit tissue-specific expression patterns [169]. When TFs cannot bind to the regulatory regions of associated genes, the methylation of cytosine in DMRs causes transcriptional inactivation or silence. Conversely, higher gene expression is linked to hypomethylation of regulatory areas [169]. The de novo DNMTs (DNMT3A, DNMT3B, and DNMT3L) and the maintenance DNA methyltransferases (DNMT1) are the names given to the DNMTs [170]. When DNA replication occurs, the enzyme DNMT1 is essential for preserving the stability of DNA methylation. Loss of methylation and abnormalities in spermatogenesis, especially on genes bearing paternal imprints, result from the lack of DNMT1. In the embryonic stage of germ cell development, DNMT3A, DNMT3B, and DNMT3L enzymes are involved in the process of DNA methylation. All DNMTs must be present to guarantee the healthy growth of sperm cells [171]. Methylation of repetitive sequences, methylation of imprinted and nonimprinted genes, and methylation of DNA on a global or genome-wide scale are among the fundamental DNA methylation activities [172]. Imprinted genes are biallelic yet exhibit themselves in a monoallelic fashion. This is because they are inherited from either the mother or the father, depending on which parent they come from. Primordial germ cells (PGCs) first experience either passive or aggressive demethylation mechanisms that demethylate their methylation markers. Subsequently, these cells exhibit variable epigenetic modifications as a result of paternal inheritance throughout the process of germline development, namely through the action of remethylation DNMTs. As the process progresses, a discernible remethylation occurs in type I spermatocytes and spermatogonia. As a result, the father's spermatozoa inherit his signature [173]. In spermatozoa, paternally imprinted/methylated genes include Igf2/H19, Rasgrfl, Dlk-Gtl2, and Zdbf2. The H19 gene is accountable for the production and transport of a 29K protein as well as RNA processing. It also encodes an untranslated cytoplasmic RNA. H19 expression is allowed and entrance from the IGF2 gene enhancer is prevented because the maternal allele of H19 DMR is unmethylated. Nevertheless, the IGF2 gene cannot express itself due to methylation of the paternal H19 DMR allele. Recently, studies on the methylation profiles of H19 DMRs and SNRPN have been conducted. In one research, oligozoospermic and azoospermic men were found to have hypomethylation of the H19 and SNRPN genes, respectively, whereas teratozoospermia and azoospermic men were found to have hypermethylation of the aforementioned genes (vs normozoospermic males) [174]. H19 gene methylation in normozoospermic men is correlated with semen parameters and ROS levels [175]. Men who are idiopathically infertile had considerably greater levels of MEST and SNRPN DMR methylation than fertile men, according to a new meta-analysis analyzing the sperm DNA methylation aberrations of imprinted genes [176]. Conversely, it was discovered that infertile guys have lower H19 DMR methylation levels than fertile men. The results of the aforementioned investigation indicate that H19 hypomethylation mostly affects sperm concentration and motility. Maternally imprinted genes include ZAC1, PEG3, SNRPN, and the mesodermal specific transcript (MEST). The expression of these genes occurs via the paternal allele due to the normal methylation of these genes in the egg and the absence of methylation in spermatozoa. The alpha/beta hydrolase family member that is encoded by the MEST gene, which is found on chromosome 7's long arm, displays paternal imprinting over the course of fetal development. MEST facilitates the alpha/beta-hydroxylase folding process, which is essential for the development of the fetal mesoderm [177].

### 6.3 Chromatin reorganization

**Fig. 4:**
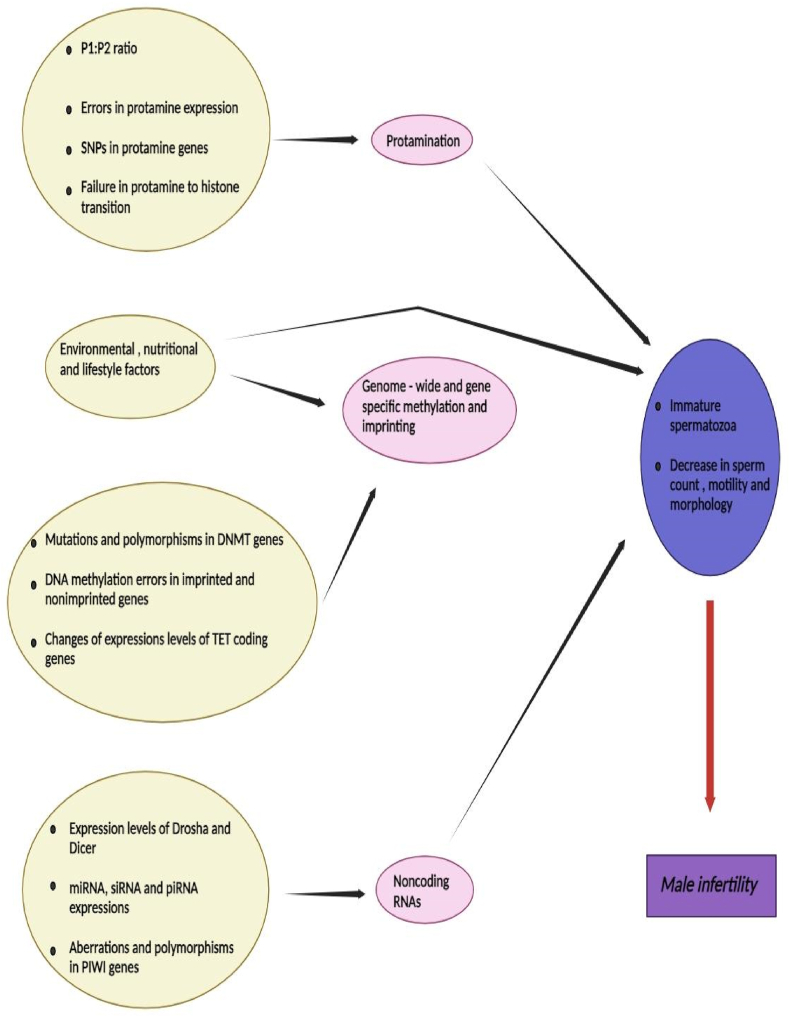
All men can become infertile and encounter reproductive diseases due to epigenetic alterations that take place during spermatogenesis.

Spermatozoa can contain massive amounts of DNA in their little nucleus due to a vital process known as chromatin rearrangement. Protonucleins are tiny proteins that are exclusive to spermatozoa. Because protamines from histones are transferred to the nucleus, sperm DNA can occupy less space there. This tight nucleus condensation boosts sperm motility. Additionally, protamination shields the sperm genome from harmful chemicals present in the female reproductive system as well as oxidation and destruction [178]. First, hyperacetylation of histone tails causes chromatin to relax and topoisomerase to stimulate breaks in DNA strands, which is the process by which protamines replace histones. Initially, transition proteins (TPs) take the place of histones. Protamines eventually fully replace transition proteins after TP1 and TP2 attach to DNA. Later on in spermatogenesis, TPs help to facilitate protamine binding to the sperm genome by aiding in the dissociation of histones [179]. It is important to remember that histones make about 5–10 % of the DNA in sperm and that these leftover histones are essential for the embryo's first growth. There is a notion stating that variations in the P1/P2 ratio or abnormalities in the protamine concentration impact the epigenetic information passed down by the paternal DNA [180]. It is crucial to understand how spermatogenesis's epigenetic processes affect male fertility (Fig. 4).

### 6.4 Histone modifications

Histone alterations can change gene expression and activity by increasing or decreasing regulatory factor affinity for DNA [181]. In testicular tissue, gene expression is enhanced by acetylation of histones H3 and H4, methylation of H3 at lysine 4 (H3K4), and ubiquitination of H2B. However, H2A ubiquitination and histone H3 methylation at lysine 9 (H3K9) and 27 (H3K27) limit the expression of some genes. Gene expression may be either repressed or promoted by methylation of H3K4 and H3K27 [182]. In both abnormal and normal human sperm samples, La Spina et al. (2014) investigated the methylation and acetylation of H3K4Ac and H4K5Ac [183]. In normal spermatozoa, researchers found different histone modifications and the markers H3K9Me2, H3K4Me1, H3K79Me2, H3K4Me3, and H3K36Me3. However, ejaculated samples with lower sperm motility had more of these markers. Yuen et al. created a mouse model with the H3f3b gene which codes for histone variant H3.3 deleted. The lack of the H3f3b gene produces faulty sperm cells, fewer germ cells, and testicular atrophy, which leads to infertility [184]. Vieweg et al. (2015) found aberrant histone acetylation of H4K12 at development-related gene promoters in sperm chromatin, indicating reduced compaction. Schon and colleagues examined normal and aberrant sperm histone post-translational modification. They observed that abnormal sperm had considerably different histone post-translational modification states than normal spermatozoa [185]. These findings show that histone modifications are crucial for sperm production and fertility. More study is needed to discover how histone tail changes affect male infertility.

### 6.5 Noncoding RNAs

Noncoding RNAs (ncRNAs) may be categorized into two primary types based on their length: long ncRNAs and short ncRNAs. The classification of ncRNAs is contingent upon their respective lengths. The three main classes of short noncoding RNAs are siRNAs, piRNAs, and miRNAs. Through epigenetic processes, these substances control the expression of genes after transcription. During spermatogenesis, male germ cells produce significant amounts of siRNAs and miRNAs. piRNAs, on the other hand, are only present in spermatocytes and round spermatids at the pachytene stage [186]. The piwi proteins are known to bind with piRNAs that consist of 30 nucleotides in length, and these interactions play a crucial role in the process of sperm maturation. The shift from histone to protamine during spermiogenesis in mice is thought to be facilitated by the piRNA machinery. The presence of azoospermia in certain people has been associated with the identification of germline mutations in the piwi gene, which have been found to impede the process of piwi ubiquitination and subsequent destruction.

## 7 Factors contributing to epigenetics changes In spermatozoa

### 7.1 Protamine abnormalities

An increase in the synthesis of immature P2 precursors has been linked to subfertility and the inability of the protamine transcription pathway to operate effectively. Though not entirely consistent, differences in the P1/P2 ratio have been linked to decreased sperm quantity and function, lower rates of fertilization and higher-quality embryos, and lower pregnancy rates [187]. However, fewer pregnancies have been associated with these findings.

### 7.2 Environmental factors

Numerous environmental factors, such as the season, the presence or absence of chemical pollutants, and an individual's lifestyle, can have a significant impact on the reproductive system [188]. 29,914 males participated in a meta-analysis assessing the impact of 13 socio-psycho-behavioral factors on semen quality [189]. A significant link was discovered in this study between the use of alcohol and a reduction in the volume of sperm. In a second large-scale investigation, semen parameters were evaluated in 11,706 males to evaluate the impact of alcohol intake, smoking, and obesity. The researchers discovered that smoking cigarettes and consuming alcohol did not affect semen parameters [190]. In a similar vein, a meta-analysis encompassing twenty research and 5,865 males revealed a correlation between exposure to cigarette smoke and decreased sperm motility, count, and morphology [191]. The authors discovered that the impact magnitude was higher in males who were infertile than in the whole population, and in those who smoked moderately to heavily than in those who smoked lightly. The relationship between lifestyle choices, food habits, exposure to environmental toxins, and epigenetic modifications such as changes to histones and noncoding RNAs and their effects on reproductive function has been the subject of recent research [192]. The researchers conducted a study in which they investigated the impact that drinking and smoking had on the quality of sperm as well as the DNA methylation patterns of repetitive DNA sequences such as GNAS, H19, LINE-1, MEST, and P16 [193]. Alterations were made to the components that were previously present in the sperm of smokers and drinkers. During a subsequent study, a 450-bead chip was utilized to investigate the response of 78 individuals who had never smoked to smoking in terms of sperm methylation at certain CpGs or genomic region patterns. The participants were either smokers or non-smokers. Researchers discovered that smoking was linked to 141 CpGs that had varying levels of methylation, which suggests that changes in environmental methylation might potentially affect the quality of sperm [194]. Some epigenetic alterations are known to take place in spermatozoa [173]. Smoking tobacco can hurt sperm epigenomic markers. The changes that follow might be passed on to the progeny and result in developmental abnormalities [195]. However, there is little information available about how smoking and drinking affect epigenetic fingerprints. More well-planned research with big cohorts and various approaches is needed.

### 7.3 Nutrition

Diet and nutrition are vital for the reproductive system to function properly [196]. Polyunsaturated fatty acids (PUFA) are found in large amounts in the membranes of sperm cells. They are needed for proper sperm activities like activation, acrosome reaction, and fusion of sperm and egg [197]. Since PUFAs cannot be produced by the body on its own, they must be received from food. PUFAs can be found in walnuts, vegetable oils, shellfish, and seeds. There are a lot of omega-3 fatty acids and docosahexaenoic acid (DHA) in these foods. Thus, dietary fatty acid intake may alter the composition of fatty acids in sperm cell membranes [198]. This is supported by research that demonstrates that consuming trans fatty acids and saturated fats, which are mostly found in meals that are fried or baked in a commercial setting, may harm the parameters of sperm, such as the number of sperm and the general quality of the sperm [199]. Research conducted on both people and animals has demonstrated that diets high in fat hurt the quality of sperm, which in turn leads to a decrease in fertility [200]. A customized diet high in fruits and vegetables is important, according to recent studies [201], and fish to raise the parameters of semen. Methylation of human DNA seems to be tightly managed, even when there are no methyl group sources in food. The presence of nutrients exerts an influence on the enzymatic activity responsible for the addition or removal of epigenetic imprints on histones and DNA. However, it is important to note that nutrients do not directly induce alterations in epigenetic marks in mice [202].

## 8 Epigenetic changing In female reproductive aging

### 8.1 DNMT-induced changes in the ageing reproductive system

There are enzymes known as DNA methyltransferases that are responsible for adding methyl groups to DNA. DNMT1 and DNMT2 are the DNMTs that are currently active in mammals. DNMT3a, DNMT3b, and DNMT3L are also active. Every single DNMT serves a unique purpose that is distinct from that of the others. Methylation of DNA may take place in two different ways: de novo methylation and maintenance methylation. Both of these modifications are possible. In the process of semi-conservative DNA replication, methylation maintenance takes place when the newly synthesized daughter strand, which is semi-unmethylated, gets methylated in the same way as the parent template strand. This occurs after the daughter strand undergoes semi-conservative methylation. This ultimately leads to the previously hemi-methylated DNA being completely methylated. The major job function of DNMT1 is the maintenance of methylation [203]. On the other hand, de novo methylation refers to the process of adding methyl groups to double-stranded DNA that already contains methyl groups. This action is carried out by DNMT3a and DNMT3b, both of which have somewhat different substrate preferences than one another [204]. While DNMT3L aids in DNMT3a and DNMT3b activity, it does not methylate DNA on its own [205]. The function of DNMT2 in the process of transfer RNA methylation is distinct [206]. In the course of normal development, the levels of various forms of DNA methyltransferase (DNMT) alter as an egg progresses from the primordial to the main and secondary follicle stages, and then from the germinal vesicle (GV) stage to MII (metaphase II) and beyond. This occurs when the egg moves from the primordial to the main and secondary follicle stages. It is at the secondary follicular stage that the expression of DNMT1 commences, and it continues throughout the zygotic stage as well as the future stages of development. The initiation of DNMT3a expression occurs during the primordial stage, whereas DNMT3b expression commences at the major follicle stage. DNMT2a is consistently undetectable at any measurable level. Preimplantation embryos have been shown to have signs of DNMT3L [207]. With each step, the position of the enzymes within the cell (cytoplasmic vs. nucleic) also varies [208]. Prior research has demonstrated a connection between developing oocytes' levels of DNMT 3a, 3b, and 3L and their maturational trajectory, as well as their participation in DNA methylation processes. This correlation implies that these enzymes have a specific function in the development of oocytes [209]. The critical role that these enzymes play in this process is further demonstrated by the similar outcomes observed in targeted gene deletions of DNMT3a or DNMT3L, which lead to embryonic death from inadequate methylation-induced imprinting failure, or the epigenetic suppression of either paternal or maternal DNA, resulting in the expression of a single chromosome for a particular trait. Notably, DNMT3b does not exhibit significant abnormalities in oocytes, suggesting that it has a secondary function throughout development [210]. As individuals become older, the regulation of DNMT changes. MII oocytes from older mice (42–45 weeks) showed changes in gene transcription related to DNA methylation compared to young mice, including increased levels of DNMT1, DNMT3L, and higher amounts of DNMT3b [211]. In addition, the research found reduced levels of DNMT3a transcription in elder mice aged 66 weeks [211]. In another study, it was demonstrated that transcription levels in human oocytes from older women were reduced in genes associated with DNA damage repair, cell cycle checkpoint, and transcription. Older mice showed a notable reduction in the levels of DNMT1, DNMT3a, DNMT3b, and DNMT3L in MII oocytes and pre-implantation embryos (2-cell, 4-cell, 8-cell, and morula) compared to younger animals. As oocytes mature, there is a gradual decrease in DNA methylation levels, which is seen in this reduction.

### 8.2 Changes in DNA methylation during reproductive ageing

Throughout an organism's lifetime, DNA methylation plays a crucial part in the evolution of development and growth, especially in germ cells and early embryos where it exhibits dynamic features [212]. Because of their highly methylated DNA, which is durable, somatic cells can express their genes in a way that is distinct from their tissues. On the other hand, sperm and mature oocytes contain similarly high amounts of methylation [208], As they mature, they go through dynamic changes. In the primordial stage, the DNA of mouse germ cells is extensively demethylated. After that, during their development from primary to secondary follicles, oocytes enter meiotic arrest and do not undergo remethylation again until after delivery [213]. Male germ cells divide continuously over a man's life and go through meiosis when he reaches maturity. Remethylation occurs in male germ cells during the prospermatagonia stage of pregnancy. After conception, the paternal and maternal chromosomes undergo different methylation changes and are physically different from one another. The maternal genome experiences passive demethylation, while the paternal genome experiences active demethylation before DNA replication [214]. Both genomes had undergone remethylation once again by the blastocyst stage, which is close to the implantation time [208]. Maintaining totipotency in the developing embryo and returning parental epigenetic changes to the germ cell DNA depends on the cycle of demethylation and remethylation. Mothers' behaviors alter as they become older. The study's findings demonstrated that compared to younger mice, older mice (35–40 weeks old) had substantially decreased DNA methylation levels. This was determined by measuring the 5-MeC fluorescence intensity in MII oocytes and preimplantation embryos at different developmental stages. Intriguingly, blastocyst DNA methylation was just minimally changed [215]. The hypothesis put up by the researchers was that this could be related to the de novo methylation that happens through DNMT3a and DNMT3b before implantation. Similar results from previous research suggested that embryos that can develop to midgestation seem to acquire and retain DNA methylation patterns normally when the mother is older than usual [216]. Additional studies were carried out to investigate the transcriptional activity of related genes and the DNA methylation levels in human granulosa cells. The granulosa cells of two different age groups of women one younger group with a mean age of 26 and the other older with a mean age of 40 were used in this study to examine the genomic DNA methylation patterns. When using assisted reproductive technology (ART), the younger group reacted to ovarian stimulation well, extracting an average of 25 oocytes, whereas the older group did not respond well, recovering an average of less than 4 oocytes. Methylated DNA Capture, Next Generation Sequencing (MethylCapseq), and Reduced Representation Bisulfite Sequencing (RRBS) were used to analyze the DNA methylome patterns between the two groups. Their findings demonstrated a more complex alteration in methylation with age. Higher-methylation regions of DNA showed rising levels of methylation with age in younger females, whereas lower-methylation regions showed falling levels of methylation with age in younger females. Therefore, as people age, their DNA methylation pattern swings even further to either extreme. Subsequently, the investigators attempted to ascertain the potential impact of this variation in methylation on gene expression. 3,397 genes were found to express differently in the two groups, many of which are related to ovarian function. Among these, 1,809 genes (such as antimullerian hormone (AMH)) had downregulation in the elderly cohort [217]. The reverse of DNA methylation is called demethylation. In mouse oocytes, it has been discovered that older mothers elevate intermediates of the demethylation cascade, such as TET enzyme levels and changed cytosines (5-hydroxymethylcytosine, 5-formylcytosine, 5-carboxylcytosine) that are the outcome of demethylation. This suggests that decreased methylation levels observed with advanced age may be caused by both increased and decreased demethylation. Moreover, demethylation-pathway-intermediates are present at different amounts in chemically accelerated aging than in normal aging, the researchers discovered [218]. This may help determine the difference between accelerated and normal aging, and possibly more significantly, assess the rate at which a woman ages reproductively and her predicted life expectancy. According to a new study, several significant genes express themselves less when maternal age causes altered DNA methylation. Researchers examined the genomic expression of human blastocysts using single embryo RNA-seq. They discovered that the expression of about 800 genes declines with maternal age. Apart from their critical roles in regulating the cell cycle and meiotic chromosomal segregation, a number of these genes might potentially be connected to aneuploidy associated with aging [219]. This underlines once more how important epigenetics is to good reproduction. It has been discovered that the oocytes of mature females exhibit changes in DNA methylation levels, DNMT levels, and histone acetylation and methylation patterns, among other enzymes linked to epigenetics (Table 4).

**Table 4:** Epigenetic modification, biological function, and different enzymes affect the oocytes.

Epigenetic modification	Biological function	Enzymes	Changes in oocytes with aging
DNA methylation	↓ transcription 3′ methylation in differentiating cells TET–demethylation ↑ transcription	DNMT–methylation	↓ DNMTs and methylation in MII oocytes and preimplantation mouse embryos [220] ↑ demethylation enzymes and intermediates in mouse oocytes [218] more extreme methylation pattern in human granulosa cells [217]
Histone ubiquitination	↑/↓ transcription (location dependent)	E1, E2, E3	–
Histone methylation	↑/↓ transcription (location dependent)	HMT–methylation KDM–demethylation	↓ in GV mouse oocytes [221] ↑ di-methylation in MII mouse oocytes [222]
Histone acetylation	↑ transcription	HDAC–deacetylation	↑ in MII mouse oocytes ↑ in MI and MII human oocytes [223]
Histone phosphorylation	↑/↓ transcription/chromatin condensation	Kinases	–
Histone sumoylation	↓ transcription	E1, E2, E3	–

### 8.3 Histone modifications in reproductive aging

Transcription is promoted by the frequent acetylation of histone N-terminal tails on lysine (K) residues [224]. Depending on where it occurs, histone methylation can either boost or reduce transcription on arginine or lysine residues. While arginine methylation on H3, H4, or H3 K4 is linked to transcription activation [225], histone H3 K9 methylation is related to transcription inhibition [226]. Histone modifications are dynamic throughout germ cell development and necessary for optimum gametogenesis, much like DNA methylation. In mouse oocytes, it has been demonstrated that during the germinal vesicle (GV) stage, histones 3, 9, and 14 are acetylated. Furthermore, lysines 5, 8, 12, and 16 of histone 4 (H3K9, H3K14, H4K5, H4K8, H4K12, and H4K16) are acetylated. During the metaphase I (MI) and metaphase II (MII) phases, HDAC deacetylates all histones except for the H4K8 histone [227]. It was found that, although having somewhat different patterns, sheep, pigs, and cows all had histone acetylation patterns that were as well-monitored [228]. On the other hand, throughout the process of oocyte development, it has been noted that histone methylation displays a high degree of stability [229]. It should be mentioned that these patterns vary according to the mother's age. Research using mouse GV oocytes revealed that compared to their younger counterparts, who had 100 % acetylation at both H4K12 and H4K16 [230], older mice had lower levels of acetylation at H4K12 and H4K16. Paradoxically, 100 % of the oocytes from young mice were found to be fully deacetylated at H4K12 during the MII stage, whereas 40 % of the oocytes from older mice were found to be acetylated at H4K12 [230]. It's interesting to note that the acetylation pattern in GV oocytes may be corrected by the researchers using the histone deacetylase inhibitor trichostatin A (TSA) to rectify the age-related acetylation anomalies. The second research looked at the H4K12 acetylation levels in early and elderly MII oocytes from the same animal to lessen genetic interactions. The older population had higher levels of H4K12 acetylation [231]. Indeed, research indicates that H4K12 acetylation levels may function as a biomarker for oocyte quality, which may impact therapeutic applications [232]. Infertility and oocyte dysfunction might result from these alterations. Research on the consequences of elevated histone acetylation in MII mouse oocytes has demonstrated that a significant incidence of oocyte aneuploidy and embryo death is linked to the suppression of histone deacetylase during meiosis. Another study used TSA to cultivate mouse oocytes to investigate the function of HDACs in meiosis. The treated oocytes experienced the same MI and MII, fertilized, and grew into blastocysts as the control group; however, they experienced a reduced incidence of implantation and a greater incidence of miscarriage. A karyotype investigation on one-cell zygotes revealed that the TSA-treated oocytes exhibited higher frequencies of hyper- and hypoploidy, which may explain their high failure rate. Inhibiting histone deacetylation resulted in higher frequencies of aneuploidy in oocytes. When the researchers assessed the H4K12 acetylation status of young and elderly oocytes, they discovered that the older group had higher levels. According to their hypothesis, this causes the chromosome to lose its capacity to take on the exact form required for accurate segregation during meiosis, which accounts for the higher incidence of aneuploidy associated with maternal age [233]. Simultaneously, another study found that in human MI and MII oocytes, aberrantly high levels of histone acetylation on H4K12 were associated with oocyte age. Older oocytes therefore had a higher likelihood of chromosomal misalignment, which increased segregation mistakes [223]. Therefore, there is substantial therapeutic significance to the elevated histone acetylation levels seen in aged oocytes during the metaphase I (MI) and metaphase II (MII) stages. Similarly, it has been shown that deacetylation of alpha-tubulin and H4K16 inside cells during meiosis promotes microtubule structure preservation and appropriate kinetochore-microtubule interactions. Alpha tubulin acetylation levels are essential for normal spindle formation and kinetochore function, just as histone acetylation. They also contribute to the spindle apparatus' stability and upkeep [234]. Increased rates of aneuploidy, chromosomal condensation and segregation abnormalities, and hyperacetylation of H4K16 were observed in mouse oocytes devoid of HDAC2 [234]. HDAC3 mutant oocytes exhibited elevated levels of tubulin acetylation and showed irregularities in spindle assembly, chromosomal alignment, kinetochore-microtubule attachments, and chromosomal mobility. The results led to a higher occurrence of myocardial infarction arrest and aneuploidy [235]. Sirtuins are a different class of cellular deacetylases. Because Sirt2 deacetylates histone H4K16 and alpha tubules, it is especially important for proper microtubule and kinetochore activity. Mice devoid of the sirt2 gene have oocytes with spindle anomalies, chromosomal instability, and a significantly decreased capacity to finish mitosis and generate a polar body. Age-related decreases in Sirt2 have been linked to greater aberrations in meiotic spindle formation and chromosomal alignment in mouse oocytes. Meiotic dysfunction rates were reduced in older oocytes that were manipulated to overexpress Sirt2 experimentally, and normal levels of H4K16 and alpha-tubulin acetylation were detected [236]. Further research looked into how aging affected oocytes' histone methylation. When comparing the GV oocytes of elderly mice to those of young mice, some documented methylation decreases in H3K9me3, H3K36me2, H3K79me2, and H4K20me2 have been found [223]. Other researchers discovered that H3K4 di- and tri-methylation was greater in the MII oocytes of the senior mice but lower in the GV oocytes of the younger animals. Moreover, it was demonstrated that the level of histone methylation affected the production of the proteins encoded by the genes encoding these changes [222]. The results emphasize the crucial impact of epigenetics on oocyte function and suggest that it may contribute to the increased aneuploidy and decreased fertility observed in older females. They also emphasize possible therapeutic methods that might decrease meiotic error and enhance patient outcomes. Research on histone phosphorylation, ubiquitination, and sumoylation in oocytes from older females has not been extensively investigated, despite evidence from gene expression studies indicating a dysregulation in genes related to ubiquitination in aged oocytes, suggesting age-related malfunction [237]. Regarding alterations in non-histone proteins in aged oocytes and potential ramifications from these changes. Histone sumoylation, for instance, is necessary for the control of mitosis and the growth of the oocyte spindle [238]. Epigenetic alterations that interfere with typical histone sumoylation processes might potentially be harmful. Additional study is required to have a more comprehensive knowledge of these alterations, particularly their occurrence in human oocytes. Further research into the mechanisms behind aging-related epigenetic modifications and the variables that might exacerbate or hasten them is also necessary [239]. Therefore, in the context of prevention, it is advisable to focus on some unique and conventional diagnostic and therapeutic techniques ([[240], [241], [242]]).

## 9 Conclusions

The issue of recurrent pregnancy loss is becoming more prevalent among young couples. A reliable and effective diagnostic method for early-stage pregnancy without a known cause has not yet been established, despite several clinical and experimental studies. Therefore, in addition to karyotyping, it is essential to identify certain genomic approaches and understand the importance of signaling pathways, clinical assessment, and pathological analysis. This has prompted research into the causes of recurrent pregnancies to improve treatment and preventative measures. The only genetic factors that are generally acknowledged to be associated with RLP are chromosomal abnormalities in the parents, which account for 2–5% of instances. The same Toll-like receptor signaling pathways may be involved in the regulation of both term labor and preterm labor brought on by infection. An increase in proinflammatory cytokine production and its effects on pregnancy-related tissues would be predicted, as these chemical messengers would initiate and maintain the labor process. Understanding TLRs and their signaling cascades in greater detail is important for both full-term and preterm labor. As human granulosa cells matured, there was a change in their methylation patterns, which was more modest. The expression of TLR at maternal-fetus contact has been characterized under both aberrant and normal situations, taking into account the location and timing of the interaction. Increasing data suggests that Toll-like receptors (TLRs) identify pathogens and trigger responses in both immune cells and non-immune cells such as trophoblasts. This points to possible therapeutic applications in pregnancy-related illnesses, such as the use of TLR expression as a diagnostic tool or the therapeutic and/or prophylactic use of TLR agonists. There are still many subjects that need explanation. While we recognize that TLRs are important for defending against infections, it is unclear how they contribute to the development of tolerance against the growing fetus. With commensal bacteria present in the reproductive canal, which may be related to the TLRs, it is intriguing to hypothesize that TLRs at the maternal interface may be important in establishing normal pregnancy; additional research is required to clarify this idea. The factors controlling TLR expression patterns and functional activity during pregnancy, whether under normal or pathological circumstances, are still not well known. Additionally, investigating this question could progress useful applications in the realm of clinical medicine. TLRs have been the subject of recent research that highlights their vital and diverse roles in several different fields. Further investigation into TLRs at the maternal-fetus interface will provide light on the processes governing the balance between the host's immune response to possible infections and tolerance towards the allergic fetus.

## Funding

There is nothing to declare.

## CRediT authorship contribution statement

**Kosar Babaei:** Writing – original draft, Methodology, Investigation. **Mohsen Azimi Nezhad:** Conceptualization. **Seyedeh Nafise Sedigh Ziabari:** Formal analysis. **Ebrahim Mirzajani:** Investigation. **Hossein Mozdarani:** Investigation. **Seyedeh Hajar Sharami:** Methodology. **Sara Farzadi:** Resources. **Seyed Reza Mirhafez:** Conceptualization. **Misa Naghdipour Mirsadeghi:** Methodology. **Seyedeh Elham Norollahi:** Writing – original draft, Investigation, Conceptualization. **Zahra Saadatian:** Writing – review & editing, Methodology, Investigation. **Ali Akbar Samadani:** Writing – review & editing, Validation, Project administration, Investigation, Data curation, Conceptualization.

## Declaration of competing interest

The authors declare that they have no known competing financial interests or personal relationships that could have appeared to influence the work reported in this paper.

## Abbreviations

IUGR: Intrauterine growth retardation
NK: Natural killer
PRRs: Pattern recognition receptors
PAMPs: Pathogen-associated molecular patterns
TLRs: Toll-like receptors
LPS: lipopolysaccharide
LTA: lipoteichoic acid
PDG: Peptidoglycan
HMGB1: High-mobility group box protein 1
EDN: Eosinophil-derived neurotoxin
Hsp: Heat-shock proteins
MyD88: Myeloid differentiation factor 88
IRF-3: IFN regulatory factor-3
HCMV: Human cytomegalovirus
IFNs: Interferons
IL-6: Interleukin-6
SLPI: Secretory leukocyte protease inhibitor
OAS: Oligoadenylate synthetase
MxA: Myxovirus resistance A
CAMs: Cell adhesion molecules
CAM: Complementary and alternative medicine
TNF-α: Tumour necrosis factor-alpha
SNPs: Single nucleotide polymorphisms
RSA: Recurrent spontaneous abortion
TGF-β: Transforming growth factor-beta
CTLA-4: Cytotoxic T-lymphocyte-associated protein 4
Tregs: Regulatory T cells
RSA: Rheumatoid arthritis
TGF-β: Growth factor-beta
Th: T helper
IFN-γ: Interferon-gamma
TNF: Tumor necrosis factor
ROS: Reactive oxygen species
DUB: Deubiquitinating enzymes
URSA: Undifferentiated rheumatoid seronegative arthritis
VEGF: Vascular endothelial growth factor
NOSs: Nitric oxide synthases
HHS: Histocompatibility Complex
HLAs: Human leukocyte antigens
IVF: In vitro fertilization
PTL: Preterm labor
DMRs: Differentially methylated regions
PGCs: Primordial germ cells
MEST: Mesodermal specific transcript
TPs: Transition proteins
H3K4: Histone H3 at lysine 4
ncRNAs: Noncoding RNAs
PUFA: Polyunsaturated fatty acids
DHA: Docosahexaenoic acid
ART: Assisted reproductive technology
RRBS: Reduced Representation Bisulfite Sequencing
AMH: Antimullerian hormone
GV: Germinal vesicle
TSA: Trichostatin A
